# A Review of Stem Cell Translation and Potential Confounds by Cancer Stem Cells

**DOI:** 10.1155/2013/241048

**Published:** 2013-12-10

**Authors:** Bernadette Bibber, Garima Sinha, Aline R. M. Lobba, Steven J. Greco, Pranela Rameshwar

**Affiliations:** ^1^Rutgers Graduate School of Biomedical and Health Sciences, Newark, NJ 07103, USA; ^2^Department of Medicine-Hematology/Oncology Rutgers, New Jersey Medical School, Newark, NJ 07103, USA; ^3^Department of Biochemistry, University of São Paulo, 05026-000 Sao Paulo, SP, Brazil; ^4^New Jersey Medical School, Rutgers School of Biomedical and Health Sciences, 185 South Orange Avenue, MSB, Room E-579, Newark, NJ 07103, USA

## Abstract

Mesenchymal stem cells (MSCs) are multipotent cells found in both fetal and adult tissues. MSCs show promise for cellular therapy for several disorders such as those associated with inflammation. In adults, MSCs primarily reside in the bone marrow (BM) and adipose tissues. In BM, MSCs are found at low frequency around blood vessels and trabecula. MSCs are attractive candidates for regenerative medicine given their ease in harvesting and expansion and their unique ability to bypass the immune system in an allogeneic host. Additionally, MSCs exert pathotropism by their ability to migrate to diseased regions. Despite the “attractive” properties of MSCs, their translation to patients requires indepth research. “Off-the-shelf” MSCs are proposed for use in an allogeneic host. Thus, the transplanted MSCs, when placed in a foreign host, could receive cue from the microenvironment for cellular transformation. An important problem with the use of MSCs involves their ability to facilitate the support of breast and other cancers as carcinoma-associated fibroblasts. MSCs could show distinct effect on each subset of cancer cells. This could lead to untoward effect during MSC therapy since the MSCs would be able to interact with undiagnosed cancer cells, which might be in a dormant state. Based on these arguments, further preclinical research is needed to ensure patient safety with MSC therapy. Here, we discuss the basic biology of MSCs, discuss current applications, and provide evidence why it is important to understand MSC biology in the context of diseased microenvironment for safe application.

## 1. Introduction

Stem cell therapy is not a new field but should be considered as an expanded field to successful bone marrow transplantation for several disorders such as autoimmune diseases and hematological malignancies. Decades of clinical application to reconstitute the hematopoietic system have led to improved methods to increase the age for transplants, resulting in benefit to an aging population [1, 2]. The long history of a focus on hematopoietic stem cells resulted in scientists overlooking other organs with tissue-specific stem cells. This past decade corrected this oversight, resulting in an “explosion” in the number of papers, journals, and scientific meetings on stem cells. The new focus correlated with an increase in registered stem cell clinical trials (clinicaltrials.gov). Those involved in the educational system across the globe are aware that stem cells are moving rapidly to the clinic while the education of future scientists and practicing physicians lags. This review discusses whether clinical trials with stem cells need a pause while scientists and a team of supporting experts become involved in robust investigational studies. We argue that such delay will ensure that stem cell delivery is done safely.

The field of stem cell provided invaluable information in cancer biology, including insights into cancer stem cells. As scientists begin to understand the latter type of stem cells, one has to ponder if undiagnosed cancer and cancer stem cells would hinder the translation of stem cell to patients. While the information on cancer stem cells is likely to lead to novel approaches to target otherwise evasive cancer cells, their “silence” or dormant phenotype existence has to be a major consideration for the safe treatment with stem cells.

Mesenchymal stem cells (MSCs) continue to show promise in cell therapy [3]. Although there are several reasons to explain why MSCs reached the clinic, a major advantage is based on the science. There is no question that embryonic stem cells (ESCs) can form any cell type. However, ESCs easily respond to *ex vivo* conditions to differentiate into different cell types. ESC “instability” poses challenges with regard to the cells' efficiency to generate a homogeneous population of a desired cell type. More importantly, ESCs can quickly form tumors when placed in an animal [4]. An attractive feature of MSCs is their ability to be used as “off-the-shelf” source for cell therapy [5], making them readily available. However, the advantages currently considered with MSCs do not give these stem cells a “green light” for absolute safety. A major issue that will be discussed in this review is the role of MSCs in cancer. Another issue with MSCs involves the culture conditions to obtain a heterogeneous population.

Despite many reports that MSCs are heterogeneous, it is difficult to determine if this occurs endogenously or if the heterogeneity is an artifact of the culture methods. This difference is an important question that needs to be addressed. Stem cell biologists will need to collaborate with biomaterial companies since they are likely to have existing “libraries” of different surfaces. Robust testing of different surfaces would determine if the type of culture method limits our ability to obtain a pure population of MSCs. However, one must be mindful that there might be an advantage to a heterogeneous population of MSCs. There is a possibility that transplanting heterogeneous MSCs in patients could be advantageous since the different cell subsets might interact to achieve a more effective response such as tissue repair. At this time, there are no “solid” experimental studies to validate the advantage of using a heterogeneous population of MSCs although this question is among many unanswered but fundamental “black boxes” in the field of stem cell biology. These questions seem to arise daily and answers are needed for effective translation to patients. One cannot help but note that the contents within requests for proposals for stem cells by funding agencies, such as the national institutes of health, do not emphasize safety issues. At this time, one wonders if the issue of safety will come to the forefront after deleterious outcomes. If so, this could slow if not end the field of translation in stem cell biology.

## 2. Mesenchymal Stem Cells (MSCs)

MSCs are in trials for different disorders (clinicaltrials.gov). In parallel, there is intense research to understand how stem cells can be translated for different disorders. MSCs can migrate towards a region of tissue injury, partly due to the expressed chemokine receptors responding to high levels of chemokines at the site of tissue damage [6].

A stem cell can differentiate into multiple lineages and undergo self-renewal. Stem cells play an important role in developmental processes, tissue repair, and protection. In recent years, the use of stem cell for several diseases such as neural and cardiac disorders has become a common theme with great promise as the future of medicine.

MSCs are adult stem cells found in human first-trimester fetal blood, liver, bone marrow, umbilical cord blood, peripheral blood, fetal membrane, placenta, adipose tissues, amniotic fluid, and multiple organs [7–11]. In adults, however, the major organs of MSCs are bone marrow and, if the individual is obese, adipose tissue. Unlike other stem cells, MSCs are easy to isolate and expand *in vitro*, whereas ESCs can form teratoma, along with the ethical issues linked to its derivation from the inner cell mass of human blastocyst [12]. Induced pluripotent stem cells (iPSCs) are derived from somatic cells through the expression of multiple genes. The iPS cells share behavior similar to ESC such as teratoma formation [13].

MSCs are spindle-shaped fibroblastoid cells. Phenotypically, MSCs express CD44, CD73, CD146, CD166, CD90, CD29, CD105/SH2/CD1-5, vimentin and endoglin, SSEA-1, and SSEA-4 [14, 15]. MSCs do not express markers associated with hematopoietic cells such as CD45 and CD34. MSCs can generate osteocyte, chondrocyte, adipocyte, myocardiocytes, neurons, hepatic cells, and bone marrow stromal cells [16–18]. We define transdifferentiation as the ability of stem cells to jump germ layer. Others argue against the transdifferentiation because it is believed that MSCs are derived from neuroectodermal cells.

MSCs interact with both innate and adaptive immune cells to exert dual immune responses, stimulation, and suppression. The type of immune response depended on the tissue microenvironment [19]. In the adult bone marrow, MSCs can be found around the blood vessel forming an interface between the periphery and the cavity [19, 20]. This location strongly suggests that MSCs could be immunologically involved in bone marrow homeostasis [21]. There are several reports stating a switch in the immune property of MSCs, depending on the level of proinflammatory cytokines. As an example, at low level, IFN-*γ* allows MSCs to be antigen presenting cells by inducing the expression of MHC-II whereas at high levels of IFN-*γ*, the MSCs switch functions to be immune suppressor [22]. MSCs can engulf foreign particles such as bacteria and, through MHC-II expression, activate T cells [23]. During the activation of T cells, IFN-*γ* levels are increased. This causes MHC-II expression to be decreased on MSCs and concomitant increase in the program cell death ligand 1 (B7-H1) to suppress the immune response [24].

IFN-*γ* also interact with other cytokines such as TNF-*α* to inhibit T-cell activation and to enhance the cytotoxic effect of natural killer (NK) cells and the proliferation and maturation of dendritic cells (DC) [25–27]. Although the exact pathways by which MSCs suppress the immune system remain an active area of investigations, the reports showed MSCs releasing cytokines such as IL-6 and IL-10 to inhibit T-cell receptor-dependent and receptor-independent proliferation of T cells. In line with the suppressive role of MSCs, these stem cells can induce and expand regulatory T cells (T_regs_), which are CD4^+^/CD25^+^. T_regs_ can act as negative regulators of inflammatory processes such as autoimmune diseases. Also, in the presence of breast cancer, MSCs can induce T_regs_ through the production of TGF-*β* [28–30].

In an experimental model of lupus, MSCs inhibit the proliferation and differentiation of B cells [31]. This occurred partly through IFN-*γ*, which activated the programmed death ligand pathway (PDL-1). The translation of the *in vitro* findings did not show a significant difference in proteinuria but showed a decrease in the deposition of glomerular immune complex. MSCs can also decrease B-cell function through the downregulation of chemokine receptors [32].

The involvement of MSCs with the innate immune system is linked to the expression of Toll-like receptor (TLR), 1–8 [33]. TLR can influence the expression of several cytokines, such as IL-6, IL-8, and IL-10 [19, 33, 34]. TLRs are single membrane noncatalytic proteins, which are important to the innate immunity [35]. TLR can activate NF*κ*B for the regulation of inflammatory cytokines [34].

**Figure 1:**
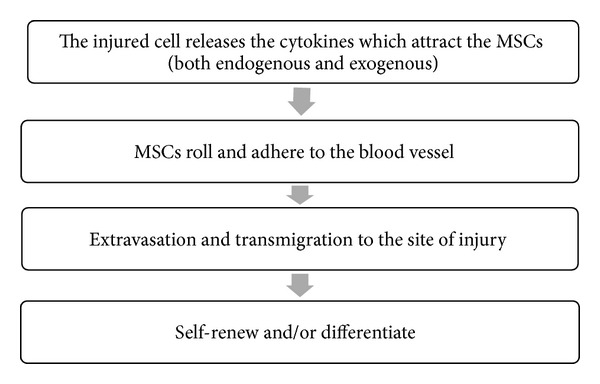
A general scheme is presented to provide an overview on the migration of MSCs to an area of tissue injury. Top row shows the release of cytokines at the region of the tissue to attract the MSCs (rows 2 and 3). Upon reaching the tissue, the MSCs can self-renew and suppress the inflammation or can differentiate to replace the damaged tissue.

Figure 1 demonstrates the basic principle of MSCs being attracted to the sites of tissue injury for tissue replacement or remaining as stem cells where they self-renew and prevent further damage. Tumor cells produce cytokines and can be considered an area of tissue damage. Thus, it is not a surprise that MSCs can also home to regions of tumors. The ability of MSCs to migrate to tumors can be explored by engineering the cells to express antitumor cytokines such as IL-12 [36]. Similarly, MSCs are being developed to transport drugs, including those within nanoparticles for brain tumors [37, 38].

## 3. MSCs-Transplantation

Clinical trials with stem cells, including those with MSCs, are registered in a national database (clinicaltrials.gov). Animal models of spinal cord injury, bone fracture, autoimmune disorder, rheumatoid arthritis, and hematopoietic defects indicated a clinical application for MSCs [6, 39–43]. The transplantation of MSCs could be from allogeneic or autologous sources. It appears that autologous transplant might pose a risk because when expanded MSCs are reintroduced into its host, the MSCs might be perceived differently from the endogenous (unexpanded) MSCs [22]. Due to the immune suppressor properties of MSCs, these cells are widely used to minimize graft versus host disease (GvHD).

The intent of transplanting MSCs for GvHD is to eventually replace or reduce the level of steroids to prevent the untoward effect associated with steroids [36]. The biggest issue with allogeneic transplantation to replace the hematopoietic system is the mismatch at the major histocompatibility complex (MHC) between the donor and recipient. As third party cells, MSCs can exert immune suppression known as veto function [44]. This property forms the basis for MSCs as third party cells to subjects receiving allogeneic bone marrow cells in transplantation. Based on the ability of MSCs to suppress allorejection, the method can be similarly applied for organ transplant. An application to suppress organ rejection will require experimental studies in large animals, which are costly but in the long-term will benefit patients and also reduce healthcare cost.

IL-10 is important during allogeneic transplantation because it inhibits IFN-*γ* production and suppresses the antigen-presenting cells, indicating that the IL-10 would prevent MSCs converting to immune-enhancing cells [23, 45]. IL-10 primed MSCs resulted in a lower mortality rate than untreated MSCs and showed significant reduction of reduced GvHD [46]. The application of MSCs for acute GvHD underscores the promise of allogeneic MSCs for stem cell therapy. Similarly, clinical trials using allogeneic MSC for acute myocardial infarct improved the patients' condition, although the mechanisms by which this occurs have not been described [5, 36]. MSCs are tested in ongoing clinical studies for neurological disorders such as Parkinson's disease and multiple sclerosis [36].

## 4. Regenerative Medicine: Other Applications

Thus far, this review mostly discussed the safety of MSCs as therapeutic use for immune suppression. However, MSCs can be used in regenerative medicine to repair damaged tissues [47]. This field represents another arm of stem cell treatment treatment in which the cells are not given to “self” but to another individual representing a different allogeneic host. Although still ongoing, this type of treatment is valuable to tissue repair to preserve organs before the damage requires transplantation. In addition to tissue regeneration, stem cells, in particular ESCs, can also be used to screen drugs.

ESCs can be induced to form any cell type such as cardiac cells. Thus, a single clone of ESC can be used to test different cell types through a rapid screening process. This will be an efficient method to prevent expensive studies and to enhance the process of getting new drugs to the clinic [48]. Although limited, ESC-derived cells are in the clinic to treat macular degeneration, heart failure, neurodegenerative disorders, and diabetes (clinicaltrials.gov). ESCs have been shown to differentiate into neural cells such as dopamine and serotonin neurons [49]. While these neurons could be used as a treatment for degenerative diseases or to repair stroke damage, there are lingering concerns with the use of ESC-derived cells. Besides the ethical reasons, these ESC-derived cells have the potential to either differentiate or dedifferentiate into the cell type that they were originally programmed to create [50].

Transplantation of hematopoietic stem cells (HSC) has been tried since 1959 to repopulate the hematopoietic system. The method has since been applied to humans for cancer, primary immunodeficiency, and other heritable and acquired diseases [51]. More recently, hematopoietic transplantation is combined with MSCs as stem cell immunotherapeutics to prevent acute GvHD [52]. Hematological malignancies such as myelomas seem to be better targeted with autologous stem cell transplant. A clinical trial with MSCs for GvHD did not show significant progress indicating additional research to effectively bring MSCs as adjuvant to transplantation [21].

The treatment of cardiac damage was tried with transplanting bone marrow mononuclear cells, which includes a mixed population of HSCs, MSCs, progenitor cells, and other hematopoietic cells [53]. Cardiac repair trials with these bone marrow cells include Bone Marrow Transfer to Enhance ST-Elevation Infarct Regeneration (BOOST) and the Transplantation of Progenitor Cells and Regeneration Enhancement to Acute Myocardial Infarction (TOPCARE-AMI). These studies resulted in improvement of the left ventricular efficiency [54–56].

To reiterate, MSCs can differentiate into different cell types such as osteocytes, chondrocytes, fibroblast, adipocytes, myocardiocytes, neurons, and hepatic cells [16–18, 57]. The differentiated cells would benefit from the field of tissue engineering for tissue repair. MSCs can also secrete factors to regulate the microenvironment to aid the tissue repair process [57]. The discussion on MSCs as immune regulators and the influence of this property to tissue repair underscores the “attractiveness” of these cells for translation to patients. Indeed, there are listed clinical trials that use MSCs for the treatment of diabetes, cirrhosis of the liver, ulcerative colitis, and spinal cord injuries (clinicaltrials.gov). In addition to bone marrow derived cells, MSCs also show promise for cardiac repair [58, 59].

## 5. Cancer Stem Cell

The cancer stem cell hypothesis proposes a small subpopulation of cancer cells that contribute to tumor formation. These cells, known as cancer stem cells (CSCs), have the ability to evade therapeutic treatments thus allowing for recurrence of cancer and metastasis [60]. Current therapies often attack the bulk of tumor cells leaving the CSCs. The surviving CSCs can continue to form new tumors or can adapt dormancy. CSCs can be caused by genetic mutations as well as other molecular changes associated with oncogenesis [61]. The method by which the CSCs survive as dormant cells and remain undetected and evade treatment is unknown. However, the experimental evidence strongly suggested that the host microenvironment and intercellular communication between the CSCs and microenvironmental cells can cause the CSCs to remain as dormant cells or cause tumors through the generation of proliferating cells and metastasis to other sites [61]. This section is included in this review because it underscores the problem that MSC treatment will encounter when they are transplanted into a subject with dormant CSCs. This issue is particularly important because MSCs can support tumor growth and also protect from the immune system. This confound is discussed below.

## 6. Breast Cancer Cells: MSC Interaction

This section further expands on CSCs of the breast since the method by which these cells adapt dormancy has been well studied. It is the goal of this review that, as MSCs progress in the clinic, the safety issue described throughout would extrapolate on the information on breast cancer for other cancers. The phenotype of breast CSCs have been well described although this is still a work in progress [62]. Breast cancer has a predilection to home and integrate to the bone marrow where they retain dormancy [63]. Once in the bone marrow, the cancer cells establish quiescence by intercellular communication with resident stromal cells [62]. Cells within the bone marrow microenvironment can also support reverse dormancy for the eventual progression and metastasis, which could partly explain resurgence [64]. MSCs, which constitute the stromal compartment of the bone marrow, can influence the migration of the cancer cells in and out of the bone marrow [64]. MSCs support the growth, invasiveness and metastatic potential of breast cancer [65–67].

## 7. Potential Confounds of MSC Treatment

There are several reports on the involvement of MSCs to support and protect solid tumors [68–70]. A major consideration when treating a patient with MSCs is undiagnosed tumor. For example, in the case of breast cancer, the experimental studies as well as the clinical evidence indicated that breast cancer cells can survive in a state of mitotic arrest for long periods as dormant cells [44, 62, 71–75]. In many cases of cancer resurgence, the bone marrow has been identified as the source of tertiary metastasis indicating the survival of initiating cancer cells in bone marrow [71–74]. This indicates that the bone marrow could be home to dormant breast cancer cells and that the original disseminated tumor cells can survive for >10 yrs [75, 76].

The heterogeneity of breast cancer cells is being developed as a hierarchy and this organizational structure is based on the relative maturation of the different subsets [62]. MSCs can induce T-cell responses such as regulatory T cells (T_regs_) to protect the cancer cells from immune cytotoxicity. The future of MSC therapy will depend on how T cells respond to the different subsets of breast cancer cells. This is important with regard to safety when MSCs are used for treatment because while they protect the tumor from the immune system through T_regs_, they can also support the growth of tumor cells [28, 77]. Breast cancer cells interact with MSCs through membrane-bound stromal cell-derived factor 1*α* (CXCL12) and its receptor, CXCR4 [78, 79]. It is unclear if all subsets interact equally. This is important in going forward to understand if stem cells, such as MSCs, should be delivered in conjunction with other agents to counteract the MSCs supporting dormant tumor cells. This brings up another point on selecting who should oversee the treatment with MSCs. In most countries, the physicians are specialized and become experts in his or her field. The field of stem cell challenges the isolationist approach. The building of teams of different subspecialty to include translational scientists would be most efficient to bring stem cells to the clinic.

As discussed above, MSCs can support tumors through increased growth and/or protect the tumor by suppressing the immune response [28, 78, 80–87]. Thus, it is important to study the immune response between MSCs and each subset of breast cancer cells. Ideally, it would be advisable to eradicate cancer and then deliver stem cells. However, at this time, there is no method to eradicate cancer, despite intense studies on the interaction between distinct cancer cell subsets and the microenvironment [88]. At this time, stem cell therapy will need to consider how the stem cells might affect the recipient who is a cancer survivor. One would assume that the surviving cancers are phenotypically stem cells. *In vivo* studies are lacking to understand how cancer stem cells interact with MSCs. Until these studies are conducted, the existence of cancer stem cells remains a liability issue for stem cell therapy. The authors of this paper do not believe that this is an easy issue for those involved in bringing stem cells to patients, but it is a fundamental problem that requires research to safely bring stem cells to patients.

Another safety issue that has little attention is the crosstalk between stem cells and molecules within the microenvironment where stem cells home and integrate. Stem cells, through specific receptors, can initiate a crosstalk with the milieu within an area of tissue injury. The site of tissue damage is likely to produce inflammatory mediators that can interact with specific receptors on the stem cells. The stem cells, in turn, would respond and produce soluble factors to activate the cells within the microenvironment [89]. Thus, it is important to understand how stem cells will respond within an area of tissue injury and whether this could be a question of safety before stem cells are given to patients.

## 8. Summary and Conclusion

This paper provides evidence to support future application of MSCs for regenerative medicine and anti-inflammatory processes. The applications for MSCs are discussed with the information that these cells could cause untoward effects. Thus, we noted a need for additional research to ensure patient safety. In particular, we cited the importance of studies to dissect the interaction between the transplanted MSCs and the tissue microenvironment. More importantly, the treatment of MSCs needs to be safe. We therefore discussed the interaction between MSCs and different subsets of breast cancer cells, in particular the cancer stem cells. MSCs can interact with cancer stem cells and support their growth. Going forward, MSC treatment will need to consider that the host may have undiagnosed cancer that could be influenced by the transplanted MSCs. We propose that parallel research studies are needed on cancer stem cells and MSCs. In summary, we propose that robust studies are needed to examine MSC biology in different diseases prior to clinical application since this will improve patient safety and increase the efficacy of stem cell treatment.

## References

[1] Chabannon C, Pamphilon D, Vermylen C (2012). Ten years after the first inspection of a candidate European centre, an EBMT registry analysis suggests that clinical outcome is improved when hematopoietic SCT is performed in a JACIE accredited program. *Bone Marrow Transplantation*.

[2] Wujcik DM (2012). As we look back at the history of BMT, we also look forward to the future of ONS Connect. *ONS Connect*.

[3] Mundra V, Gerling IC, Mahato RI (2013). Mesenchymal stem cell-based therapy. *Molecular Pharmaceutics*.

[4] Peng X, Liu T, Wang Y (2012). Wnt/beta-catenin signaling in embryonic stem cell converted tumor cells. *Journal of Translational Medicine*.

[5] Hare JM, Fishman JE, Gerstenblith : G (2012). Comparison of allogeneic vs autologous bone marrow-derived mesenchymal stem cells delivered by transendocardial injection in patients with ischemic cardiomyopathy: the poseidon randomized trial. *Journal of the American Medical Association*.

[6] Chen FM, Wu LA, Zhang M, Zhang R, Sun HH (2011). Homing of endogenous stem/progenitor cells for in situ tissue regeneration: promises, strategies, and translational perspectives. *Biomaterials*.

[7] Campagnoli C, Roberts IAG, Kumar S, Bennett PR, Bellantuono I, Fisk NM (2001). Identification of mesenchymal stem/progenitor cells in human first-trimester fetal blood, liver, and bone marrow. *Blood*.

[8] He Q, Wan C, Li G (2007). Concise review: multipotent mesenchymal stromal cells in blood. *Stem Cells*.

[9] Lee OK, Kuo TK, Chen WM, Lee KD, Hsieh SL, Chen TH (2004). Isolation of multipotent mesenchymal stem cells from umbilical cord blood. *Blood*.

[10] Romanov YA, Svintsitskaya VA, Smirnov VN (2003). Searching for alternative sources of postnatal human mesenchymal stem cells: candidate MSC-like cells from umbilical cord. *Stem Cells*.

[11] Tsuda H, Yamahara K, Ishikane S (2010). Allogenic fetal membrane-derived mesenchymal stem cells contribute to renal repair in experimental glomerulonephritis. *American Journal of Physiology—Renal Physiology*.

[12] Thomson JA (1998). Embryonic stem cell lines derived from human blastocysts. *Science*.

[13] Gutierrez-Aranda I, Ramos-Mejia V, Bueno C (2010). Human induced pluripotent stem cells develop teratoma more efficiently and faster than human embryonic stem cells regardless the site of injection. *Stem Cells*.

[14] Anjos-Afonso F, Bonnet D (2007). Nonhematopoietic/endothelial SSEA-1+ cells define the most primitive progenitors in the adult murine bone marrow mesenchymal compartment. *Blood*.

[15] Gang EJ, Bosnakovski D, Figueiredo CA, Visser JW, Perlingeiro RCR (2007). SSEA-4 identifies mesenchymal stem cells from bone marrow. *Blood*.

[16] Bonfield TL, Caplan AI (2010). Adult mesenchymal stem cells: an innovative therapeutic for lung diseases. *Discovery medicine*.

[17] Tang QQ, Otto TC, Lane MD (2004). Commitment of C3H10T1/2 pluripotent stem cells to the adipocyte lineage. *Proceedings of the National Academy of Sciences of the United States of America*.

[18] Zhang X, Schwarz EM, Young DA, Edward Puzas J, Rosier RN, O’Keefe RJ (2002). Cyclooxygenase-2 regulates mesenchymal cell differentiation into the osteoblast lineage and is critically involved in bone repair. *Journal of Clinical Investigation*.

[19] Yagi H, Soto-Gutierrez A, Parekkadan B (2010). Mesenchymal stem cells: mechanisms of immunomodulation and homing. *Cell Transplantation*.

[20] Bianco P, Riminucci M, Gronthos S, Robey PG (2001). Bone marrow stromal stem cells: nature, biology, and potential applications. *Stem Cells*.

[21] Rameshwar P (2008). IFN*γ* and B7-H1 in the immunology of mesenchymal stem cells. *Cell Research*.

[22] Patel SA, Sherman L, Munoz J, Rameshwar P (2008). Immunological properties of mesenchymal stem cells and clinical implications. *Archivum Immunologiae et Therapiae Experimentalis*.

[23] Chan JL, Tang KC, Patel AP (2006). Antigen-presenting property of mesenchymal stem cells occurs during a narrow window at low levels of interferon-*γ*. *Blood*.

[24] Sheng H, Wang Y, Jin Y (2008). A critical role of IFN*γ* in priming MSC-mediated suppression of T cell proliferation through up-regulation of B7-H1. *Cell Research*.

[25] Bartholomew A, Sturgeon C, Siatskas M (2002). Mesenchymal stem cells suppress lymphocyte proliferation in vitro and prolong skin graft survival in vivo. *Experimental Hematology*.

[26] Jiang XX, Zhang Y, Liu B (2005). Human mesenchymal stem cells inhibit differentiation and function of monocyte-derived dendritic cells. *Blood*.

[27] Nauta AJ, Kruisselbrink AB, Lurvink E, Willemze R, Fibbe WE (2006). Mesenchymal stem cells inhibit generation and function of both CD34 +-derived and monocyte-derived dendritic cells. *Journal of Immunology*.

[28] Patel SA, Meyer JR, Greco SJ, Corcoran KE, Bryan M, Rameshwar P (2010). Mesenchymal stem cells protect breast cancer cells through regulatory T cells: role of mesenchymal stem cell-derived TGF-*β*. *Journal of Immunology*.

[29] Jonuleit H, Schmitt E (2003). The regulator T cell family: distinct subsets and their interrelations. *Journal of Immunology*.

[30] Suri-Payer E, Amar AZ, Thornton AM, Shevach EM (1998). CD4+CD25+ T cells inhibit both the induction and effector function of autoreactive T cells and represent a unique lineage of immunoregulatory cells. *Journal of Immunology*.

[31] Schena F, Gambini C, Gregorio A (2010). Interferon-*γ*-dependent inhibition of B cell activation by bone marrow-derived mesenchymal stem cells in a murine model of systemic lupus erythematosus. *Arthritis and Rheumatism*.

[32] Corcione A, Benvenuto F, Ferretti E (2006). Human mesenchymal stem cells modulate B-cell functions. *Blood*.

[33] Pevsner-Fischer M, Morad V, Cohen-Sfady M (2007). Toll-like receptors and their ligands control mesenchymal stem cell functions. *Blood*.

[34] Liotta F, Angeli R, Cosmi L (2008). Toll-like receptors 3 and 4 are expressed by human bone marrow-derived mesenchymal stem cells and can inhibit their T-cell modulatory activity by impairing notch signaling. *Stem Cells*.

[35] Nagai Y, Garrett KP, Ohta S (2006). Toll-like receptors on hematopoietic progenitor cells stimulate innate immune system replenishment. *Immunity*.

[36] Ren G, Chen X, Dong F (2012). Concise review: mesenchymal stem cells and translational medicine: emerging issues. *Stem Cells Translational Medicine*.

[37] Cheng H, Kastrup CJ, Ramanathan R (2010). Nanoparticulate cellular patches for cell-mediated tumoritropic delivery. *ACS Nano*.

[38] Roger M, Clavreul A, Venier-Julienne MC (2010). Mesenchymal stem cells as cellular vehicles for delivery of nanoparticles to brain tumors. *Biomaterials*.

[39] Constantin G, Marconi S, Rossi B (2009). Adipose-derived mesenchymal stem cells ameliorate chronic experimental autoimmune encephalomyelitis. *Stem Cells*.

[40] Hofstetter CP, Schwarz EJ, Hess D (2002). Marrow stromal cells form guiding strands in the injured spinal cord and promote recovery. *Proceedings of the National Academy of Sciences of the United States of America*.

[41] Kitaori T, Ito H, Schwarz EM (2009). Stromal cell-derived factor 1/CXCR4 signaling is critical for the recruitment of mesenchymal stem cells to the fracture site during skeletal repair in a mouse model. *Arthritis and Rheumatism*.

[42] Marinova-Mutafchieva L, Taylor P, Funa K, Maini RN, Zvaifler NJ (2000). Mesenchymal cells expressing bone morphogenetic protein receptors are present in the rheumatoid arthritis joint. *Arthritis & Rheumatism*.

[43] Quarto R, Mastrogiacomo M, Cancedda R (2001). Repair of large bone defects with the use of autologous bone marrow stromal cells. *New England Journal of Medicine*.

[44] Rao G, Patel PS, Idler SP (2004). Facilitating role of preprotachykinin-I dene in the integration of breast cancer cells within the stromal compartment of the bone marrow: a model of early cancer progression. *Cancer Research*.

[45] Ge X, Bai C, Yang J, Lou G, Li Q, Chen R (2013). Intratracheal transplantation of bone marrow-derived mesenchymal stem cells reduced airway inflammation and up-regulated CD4(+)CD25(+) regulatory T cells in asthmatic mouse. *Cell Biol Int*.

[46] Min CK, Kim BG, Park G, Cho B, Oh IH (2007). IL-10-transduced bone marrow mesenchymal stem cells can attenuate the severity of acute graft-versus-host disease after experimental allogeneic stem cell transplantation. *Bone Marrow Transplantation*.

[47] Collins FS, Haseltine WA (2000). Of genes and genomes: what lies between the base pairs. *Journal of Investigative Medicine*.

[48] He JQ, Ma Y, Lee Y, Thomson JA, Kamp TJ (2003). Human embryonic stem cells develop into multiple types of cardiac myocytes: action potential characterization. *Circulation Research*.

[49] Björklund LM, Sánchez-Pernaute R, Chung S (2002). Embryonic stem cells develop into functional dopaminergic neurons after transplantation in a Parkinson rat model. *Proceedings of the National Academy of Sciences of the United States of America*.

[50] Suzuki A, Raya Á, Kawakami Y (2006). Maintenance of embryonic stem cell pluripotency by Nanog-mediated reversal of mesoderm specification. *Nature Clinical Practice Cardiovascular Medicine*.

[51] Hymes SR, Alousi AM, Cowen EW (2012). Graft-versus-host disease. Part II: management of cutaneous graft-versus-host disease. *Journal of the American Academy of Dermatology*.

[52] Papewalis C, Topolar D, Gotz B, Schonberger S, Dilloo : D (2013). Mesenchymal stem cells as cellular immunotherapeutics in allogeneic hematopoietic stem cell transplantation. *Advances in Biochemical Engineering/Biotechnology*.

[53] Boyle AJ, Schulman SP, Hare JM (2006). Stem cell therapy for cardiac repair: ready for the next step. *Circulation*.

[54] Assmus B, Schächinger V, Teupe C (2002). Transplantation of progenitor cells and regeneration enhancement in acute myocardial infarction (TOPCARE-AMI). *Circulation*.

[55] Schächinger V, Assmus B, Britten MB (2004). Transplantation of progenitor cells and regeneration enhancement in acute myocardial infarction: final one-year results of the TOPCARE-AMI trial. *Journal of the American College of Cardiology*.

[56] Wollert KC, Meyer GP, Lotz J (2004). Intracoronary autologous bone-marrow cell transfer after myocardial infarction: the BOOST randomised controlled clinical trial. *Lancet*.

[57] Caplan AI (2007). Adult mesenchymal stem cells for tissue engineering versus regenerative medicine. *Journal of Cellular Physiology*.

[58] Lim PK, Bliss SA, Patel SA (2011). Gap junction-mediated import of microRNA from bone marrow stromal cells can elicit cell cycle quiescence in breast cancer cells. *Cancer Research*.

[59] Min JY, Sullivan MF, Yang Y (2002). Significant improvement of heart function by cotransplantation of human mesenchymal stem cells and fetal cardiomyocytes in postinfarcted pigs. *Annals of Thoracic Surgery*.

[60] Delude C (2011). Tumorigenesis: testing ground for cancer stem cells. *Nature*.

[61] Greaves M, Maley CC (2012). Clonal evolution in cancer. *Nature*.

[62] Patel SA, Ramkissoon SH, Bryan M (2012). Delineation of breast cancer cell hierarchy identifies the subset responsible for dormancy. *Scientific Reports*.

[63] Goldstein RH, Reagan MR, Anderson K, Kaplan DL, Rosenblatt M (2010). Human bone marrow-derived mscs can home to orthotopic breast cancer tumors and promote bone metastasis. *Cancer Research*.

[64] Reddy BY, Lim PK, Silverio K, Patel SA, Won BW, Rameshwar P (2012). The microenvironmental effect in the progression, metastasis, and dormancy of breast cancer: a model system within bone marrow. *International Journal of Breast Cancer*.

[65] Karnoub AE, Dash AB, Vo AP (2007). Mesenchymal stem cells within tumour stroma promote breast cancer metastasis. *Nature*.

[66] Chaturvedi P, Gilkes DM, Wong CCL (2013). Hypoxia-inducible factor-dependent breast cancer-mesenchymal stem cell bidirectional signaling promotes metastasis. *Journal of Clinical Investigation*.

[67] Yan XL, Fu CJ, Chen L (2012). Mesenchymal stem cells from primary breast cancer tissue promote cancer proliferation and enhance mammosphere formation partially via EGF/EGFR/Akt pathway. *Breast Cancer Research and Treatment*.

[68] Bergfeld SA, DeClerck YA (2010). Bone marrow-derived mesenchymal stem cells and the tumor microenvironment. *Cancer and Metastasis Reviews*.

[69] Reagan MR, Kaplan DL (2011). Concise review: mesenchymal stem cell tumor-homing: detection methods in disease model systems. *Stem Cells*.

[70] Torsvik A, Bjerkvig R (2013). Mesenchymal stem cell signaling in cancer progression. *Cancer Treatment Reviews*.

[71] Habeck M (2000). Bone-marrow analysis predicts breast-cancer recurrence. *Molecular Medicine Today*.

[72] Mansi JL, Berger U, McDonnell T (1989). The fate of bone marrow micrometastases in patients with primary breast cancer. *Journal of Clinical Oncology*.

[73] Naume B, Zhao X, Synnestvedt M (2007). Presence of bone marrow micrometastasis is associated with different recurrence risk within molecular subtypes of breast cancer. *Molecular Oncology*.

[74] Riethdorf S, Wikman H, Pantel K (2008). Review: biological relevance of disseminated tumor cells in cancer patients. *International Journal of Cancer*.

[75] Klein CA (2011). Framework models of tumor dormancy from patient-derived observations. *Current Opinion in Genetics and Development*.

[76] Aguirre-Ghiso JA, Bragado P, Sosa MS (2013). Metastasis awakening: targeting dormant cancer. *Nature Medicine*.

[77] Yamaguchi T, Wing JB, Sakaguchi S (2011). Two modes of immune suppression by Foxp3+ regulatory T cells under inflammatory or non-inflammatory conditions. *Seminars in Immunology*.

[78] Corcoran KE, Trzaska KA, Fernandes H (2008). Mesenchymal stem cells in early entry of breast cancer into bone marrow. *PLoS ONE*.

[79] Greco SJ, Patel SA, Bryan M, Pliner LF, Banerjee D, Rameshwar P (2011). AMD3100-mediated production of interleukin-1 from mesenchymal stem cells is key to chemosensitivity of breast cancer cells. *American Journal of Cancer Research*.

[80] Comşa Ş, Ciuculescu F, Raica M (2012). Mesenchymal stem cell-tumor cell cooperation in breast cancer vasculogenesis. *Molecular Medicine Reports*.

[81] Momin EN, Vela G, Zaidi HA, Quiñones-Hinojosa A (2010). The oncogenic potential of mesenchymal stem cells in the treatment of cancer: directions for future research. *Current Immunology Reviews*.

[82] Feng B, Chen L (2009). Review of mesenchymal stem cells and tumors: executioner or coconspirator?. *Cancer Biotherapy and Radiopharmaceuticals*.

[83] Rameshwar P (2010). Breast cancer cell dormancy in bone marrow: potential therapeutic targets within the marrow microenvironment. *Expert Review of Anticancer Therapy*.

[84] Greco SJ, Rameshwar P (2008). Microenvironmental considerations in the application of human mesenchymal stem cells in regenerative therapies. *Biologics: Targets and Therapy*.

[85] Riggi N, Suvà ML, de Vito C (2010). EWS-FLI-1 modulates miRNA145 and SOX2 expression to initiate mesenchymal stem cell reprogramming toward Ewing sarcoma cancer stem cells. *Genes and Development*.

[86] de Boeck A, Narine K, de Neve W, Mareel M, Bracke M, de Wever O (2010). Resident and bone marrow-derived mesenchymal stem cells in head and neck squamous cell carcinoma. *Oral Oncology*.

[87] Mishra PJ, Mishra PJ, Humeniuk R (2008). Carcinoma-associated fibroblast-like differentiation of human mesenchymal stem cells. *Cancer Research*.

[88] Badve S, Nakshatri H (2012). Breast-cancer stem cells-beyond semantics. *The Lancet Oncology*.

[89] Liu X, Duan B, Cheng Z (2011). SDF-1/CXCR4 axis modulates bone marrow mesenchymal stem cell apoptosis, migration and cytokine secretion. *Protein and Cell*.

